# Supplementary material for: Analysis of PPARγ Signaling Activity in Psoriasis
Source: Int J Mol Sci. 2021 Aug 10;22(16):8603. doi: 10.3390/ijms22168603 (PMC8395241; doi:10.3390/ijms22168603)
Supplement: Supplementary file 1 [file ijms-22-08603-s001.zip › Supplemental materials_Analysis of PPARg signaling activity in psoriasis/Pathway models/Models images and html files/Differentiation of psoriatic T cells/13714.html]

SOCS3


# Protein SOCS3

|  |  |
| --- | --- |
| URN | urn:agi-llid:9021 |
| Total Entities | 0 |
| Connectivity | 3359 |
| Name | SOCS3 |
| Description | suppressor of cytokine signaling 3 |
| Notes | This gene encodes a member of the STAT-induced STAT inhibitor (SSI), also known as suppressor of cytokine signaling (SOCS), family. SSI family members are cytokine-inducible negative regulators of cytokine signaling. The expression of this gene is induced by various cytokines, including IL6, IL10, and interferon (IFN)-gamma. The protein encoded by this gene can bind to JAK2 kinase, and inhibit the activity of JAK2 kinase. Studies of the mouse counterpart of this gene suggested the roles of this gene in the negative regulation of fetal liver hematopoiesis, and placental development. [provided by RefSeq, Jul 2008] |
| Primary Cell Localization | Cytoplasm |

---

|  |  |
| --- | --- |
| Pathway | Leptin and CCK8 Activate Nodose Ganglia Neuron |
|  | AXL Receptor Inhibits Macrophages and Dendritic Cells Function |
|  | Proteins Involved in Atherosclerosis |
|  | SOCS in Negative Feedback Regulation in Myocarditis |
|  | Proteins Involved in Melanoma |
|  | IFN-gamma/TNF-alpha Mediated Cell Proliferation in Psoriasis |
|  | Proteins with Altered Expression in Psoriasis |
|  | Proteins Involved in Diabetes Mellitus Type 2 |
|  | Adipokines and Cytokines in Insulin Resistance in Skeletal Muscles |
|  | Proteins Involved in Insulin Resistance |
|  | Insulin Resistance in Myocytes Induced by Obesity |
|  | Insulin Resistance in Hepatocytes |
|  | Thyroid Hormones in Adipose Tissue Metabolism |
|  | SOCS3 and PTPN1 in Hypothalamic Neuron Insensitivity to Insulin and Leptin |
|  | Proteins Involved in Obesity |
|  | Proteins Involved in Ulcerative Colitis |
|  | Proteins Involved in Follicular Lymphoma |
|  | TRPM7/8 in Pancreatic Adenocarcinoma |
|  | IL6 and Insulin Resistance in Muscular Dystrophies |
|  | IL6 Promotes Inflammation in Muscular Dystrophies |
|  | Proteins Involved in Glioblastoma |
|  | Proteins Involved in Endometriosis |
|  | IL10/STAT3 Signaling in M2 Macrophage and Retinal Angiogenesis |
|  | Proteins with Altered Expression in Age-Related Macular Degeneration |
|  | Alveolar Macrophage Negative Regulation Declined in COPD |
|  | Noradrenaline/Gs Expression Targets |
|  | Noreadrenaline/Gq Expression Targets |
|  | Epinephrine/Gs Expression Targets |
|  | Epinephrine/Gq Expression Targets |
|  | CLCF1 Expression Targets |
|  | CCL15 Expression Targets |
|  | CSF3 Expression Targets |
|  | LIF Expression Targets |
|  | CNTF Expression Targets |
|  | EGF -> CTNN Expression Targets |
|  | EGF -> AP-1/ATF Expression Targets |
|  | EGF -> CREB/CREBBP/ELK/SRF/MYC Expression Targets |
|  | EGF -> MEF/MYOD/NFATC Expression Targets |
|  | EGF -> STAT Expression Targets |
|  | F2 -> AP-1/CREB/ELK/SRF/SP1 Expression Targets |
|  | F2 -> STAT1/NF-kB Expression Targets |
|  | FGF2 -> STAT Expression Targets |
|  | FGF2 -> AP-1/CREB/CREBBP/ELK/SRF/MYC Expression Targets |
|  | GCG Expression Targets |
|  | ADCYAP1 Expression Targets |
|  | GH1/GHR -> NF-kB/ELK/SRF/MYC Expression Targets |
|  | GH1/GHR -> STAT Expression Targets |
|  | GH1/PRLR Expression Targets |
|  | HBEGF -> STAT Expression Targets |
|  | HBEGF -> FOXO3A Expression Target |
|  | HBEGF -> AP-1/ATF Expression Targets |
|  | HGF -> STAT Expression Targets |
|  | HGF -> AP-1/CREB/ELK/SRF/MYC Expression Targets |
|  | IGF1 -> MEF/MYOD/MYOG Expression Targets |
|  | IGF1 -> STAT Expression Targets |
|  | IGF1 -> ELK/SRF/HIF1A/MYC/SREBF Expression Targets |
|  | INHBA/ACVR2/BMPR Expression Targets |
|  | Insulin -> STAT Expression Targets |
|  | Insulin -> CEBPA/CTNNB/FOXA/FOXO Expression Targets |
|  | Insulin -> MEF/MYOD Expression Targets |
|  | Insulin -> ELK/SRF/HIF1A/MYC/SREBF Expression Targets |
|  | EGFR -> Expression Targets in Skin |
|  | IFNA1/IFNR Expression Targets |
|  | IFNB1/IFNR Expression Targets |
|  | IFNG/IFNR Expression Targets |
|  | IL21 Expression Targets |
|  | IL15 Expression Targets |
|  | IL6 Expression Targets |
|  | IL10 Expression Targets |
|  | IL22 Expression Targets |
|  | IL11 Expression Targets |
|  | IL9 Expression Targets |
|  | IL4 Expression Targets |
|  | Leptin -> STAT Expression Targets |
|  | Leptin -> ELK/SRF Expression Targets |
|  | Elevated Receptors -> Expression Targets in Lymphoid System and Blood |
|  | F2R/F2RL1 -> Expression Targets in Lymphoid System and Blood |
|  | Elevated Receptors -> Expression Targets in Nerve Tissue |
|  | DLL4 Expression Targets |
|  | DLL1 Expression Targets |
|  | NOTCH Expression Targets |
|  | OSM/IL6ST/LIFR Expression Targets |
|  | PDGF -> AP-1/CREB/CREBBP/MYC Expression Targets |
|  | PDGF -> STAT Expression Targets |
|  | AGT -> CREB Expression Targets |
|  | IFNA1/Gq Expression Targets |
|  | AGT -> STAT Expression Targets |
|  | PRL/PRLR Expression Targets |
|  | PRL/GHR -> STAT Expression Targets |
|  | PRL/GHR -> NF/kB/ELK/SRF/MYC Expression Targets |
|  | Elevated Receptors -> Expression Targets in Lung |
|  | Ras-GAP Regulation Signaling |
|  | AXL Receptor Tyrosine Kinase Signaling |
|  | TCR -> STAT Expression Targets |
|  | TCR -> AP-1 Expression Targets |
|  | TGFB1-TGFBR2 Expression Targets |
|  | TGFB1-TGFBR1/AP-1 Expression Targets |
|  | TGFB1-ACVRL1 Expression Targets |
|  | TNF -> NF-kB Expression Targets |
|  | TNFSF14 Expression Targets |
|  | TNF -> AP-1 Expression Targets |
|  | TNF -> STAT Expression Targets |
|  | Thrombopoietin -> AP-1/CREB/CREBBP/MYC Expression Targets |
|  | Thrombopoietin -> STAT Expression Targets |
|  | Thrombopoietin -> SP1 Expression Targets |
|  | TLR4 -> AP-1 Expression Targets |
|  | IL1B -> PGE2 Expression Targets |
|  | IL1B Expression Targets |
|  | VEGFA -> ATF/CREB/ELK-SRF Expression Targets |
|  | VEGFA -> STAT Expression Targets |
|  | VEGFA -> AP-1/CREBBP/MYC Expression Targets |
|  | VEGFA -> CTNNB/CTNND Expression Targets |
|  | ace2 |
|  | prarg neg,ukn expres targets, ps-positive |
|  | PPARG negative targets |
|  | pparg expr target\_regulators |
|  | Neighbors of disease exacerbation |
|  | Neighbors of tumor progression |
|  | Neighbors of cancer progression |
|  | Neighbors of overall survival |
|  | Neighbors of disease severity |
|  | Neighbors of tumor size |
|  | Neighbors of lesion size |
|  | Neighbors of mortality |
|  | Neighbors of survival rate |
|  | Neighbors of infarct size |
|  | Neighbors of survival time |
|  | Neighbors of functional recovery |
|  | Neighbors of insulin sensitivity |
|  | Neighbors of graft survival |
|  | Neighbors of therapeutic efficacy |
|  | Neighbors of clinical stage |
|  | Neighbors of lethality |
|  | Neighbors of recurrence-free survival |
|  | Neighbors of body weight |
|  | Neighbors of body weight gain |
|  | Neighbors of neutrophil count |
|  | Neighbors of glucose tolerance |
|  | Neighbors of fibrosis progression |
|  | Neighbors of fat mass |
|  | Neighbors of exhaustion |
|  | Neighbors of radioresistance |
|  | Neighbors of apoptosis rate |
|  | Neighbors of early mortality |
|  | Neighbors of viral load |
|  | Neighbors of blood pressure |
|  | Neighbors of leukocyte count |
|  | Neighbors of muscle mass |
|  | Neighbors of blood glucose |
|  | Neighbors of fasting plasma glucose |
|  | Neighbors of osteoclast number |
|  | Neighbors of energy expenditure |
|  | Neighbors of glucose level |
|  | Neighbors of radiosensitivity |
|  | Neighbors of pregnancy outcome |
|  | Neighbors of functional status |
|  | Neighbors of neurological outcome |
|  | Neighbors of infarct area |
|  | Neighbors of fertility |
|  | Neighbors of insulin level |
|  | Neighbors of disease exacerbation |
|  | Neighbors of tumor progression |
|  | Neighbors of cancer progression |
|  | Neighbors of overall survival |
|  | Neighbors of disease severity |
|  | Neighbors of tumor size |
|  | Neighbors of lesion size |
|  | Neighbors of mortality |
|  | Neighbors of survival rate |
|  | Neighbors of infarct size |
|  | Neighbors of survival time |
|  | Neighbors of functional recovery |
|  | Neighbors of insulin sensitivity |
|  | Neighbors of graft survival |
|  | Neighbors of therapeutic efficacy |
|  | Neighbors of clinical stage |
|  | Neighbors of lethality |
|  | Neighbors of recurrence-free survival |
|  | Neighbors of body weight |
|  | Neighbors of body weight gain |
|  | Neighbors of neutrophil count |
|  | Neighbors of glucose tolerance |
|  | Neighbors of fibrosis progression |
|  | Neighbors of fat mass |
|  | Neighbors of exhaustion |
|  | Neighbors of radioresistance |
|  | Neighbors of apoptosis rate |
|  | Neighbors of early mortality |
|  | Neighbors of viral load |
|  | Neighbors of blood pressure |
|  | Neighbors of leukocyte count |
|  | Neighbors of muscle mass |
|  | Neighbors of blood glucose |
|  | Neighbors of fasting plasma glucose |
|  | Neighbors of osteoclast number |
|  | Neighbors of energy expenditure |
|  | Neighbors of glucose level |
|  | Neighbors of radiosensitivity |
|  | Neighbors of pregnancy outcome |
|  | Neighbors of functional status |
|  | Neighbors of neurological outcome |
|  | Neighbors of infarct area |
|  | Neighbors of fertility |
|  | Neighbors of insulin level |
|  | Neighbors of cell development |
|  | Neighbors of immune response |
|  | Neighbors of cell population |
|  | Neighbors of T-cell development |
|  | Neighbors of inflammatory response |
|  | Neighbors of wound healing |
|  | Neighbors of angiogenesis |
|  | Neighbors of innate immune response |
|  | Neighbors of cell function |
|  | Neighbors of macrophage activation |
|  | Neighbors of cell formation |
|  | Neighbors of adaptive immune response |
|  | Neighbors of osteoclast development |
|  | Neighbors of epithelial to mesenchymal transition |
|  | Neighbors of immunity |
|  | Neighbors of cell proliferative response |
|  | Neighbors of chemotaxis |
|  | Neighbors of dendritic cell differentiation |
|  | Neighbors of cell survival |
|  | Neighbors of osteoclast differentiation |
|  | Neighbors of cell phenotype |
|  | Neighbors of T-cell proliferation |
|  | Neighbors of cell adhesion |
|  | Neighbors of T-cell activation |
|  | Neighbors of tumor growth |
|  | Neighbors of dendritic cell development |
|  | Neighbors of tissue repair |
|  | Neighbors of cell count |
|  | Neighbors of cell infiltration |
|  | Neighbors of SMC proliferation |
|  | Neighbors of lumen formation |
|  | Neighbors of adipogenesis |
|  | Neighbors of lipid storage |
|  | Neighbors of cell damage |
|  | Neighbors of pregnancy |
|  | Neighbors of aging |
|  | Neighbors of cell motility |
|  | Neighbors of fibroblast proliferation |
|  | Neighbors of microglial activation |
|  | Neighbors of neutrophil recruitment |
|  | Neighbors of regeneration |
|  | Neighbors of cell migration |
|  | Neighbors of cancer growth |
|  | Neighbors of stem cell proliferation |
|  | Neighbors of smooth muscle cell migration |
|  | Neighbors of hepatic regeneration |
|  | Neighbors of phagocytosis |
|  | Neighbors of immune system activation |
|  | Neighbors of leukocyte migration |
|  | Neighbors of osteoclast formation |
|  | Neighbors of monocyte recruitment |
|  | Neighbors of cell invasion |
|  | Neighbors of heart function |
|  | Neighbors of hemopoiesis |
|  | Neighbors of bone remodeling |
|  | Neighbors of ossification |
|  | Neighbors of T-cell function |
|  | Neighbors of immune system function |
|  | Neighbors of lung development |
|  | Neighbors of autophagy |
|  | Neighbors of monocyte adhesion |
|  | Neighbors of transcription activation |
|  | Neighbors of senescence |
|  | Neighbors of T-cell response |
|  | Neighbors of tumor immunity |
|  | Neighbors of hepatocyte proliferation |
|  | Neighbors of cell transdifferentiation |
|  | Neighbors of neuroprotection |
|  | Neighbors of stem cell differentiation |
|  | Neighbors of endothelial cell function |
|  | Neighbors of neuronal death |
|  | Neighbors of cell growth |
|  | Neighbors of ROS generation |
|  | Neighbors of epithelial cell proliferation |
|  | Neighbors of cancer cell growth |
|  | Neighbors of sensitization |
|  | Neighbors of macrophage function |
|  | Neighbors of cellular senescence |
|  | Neighbors of colony formation |
|  | Neighbors of macrophage differentiation |
|  | Neighbors of cell differentiation |
|  | Neighbors of keratinocyte proliferation |
|  | Neighbors of cell homeostasis |
|  | Neighbors of psoriasis |
|  | Neighbors of inflammatory disease |
|  | Neighbors of rheumatoid arthritis |
|  | Neighbors of atherosclerosis |
|  | Neighbors of fibrosis |
|  | Neighbors of colitis |
|  | Neighbors of arthritis |
|  | Neighbors of inflammatory bowel disease |
|  | Neighbors of injury |
|  | Neighbors of metastasis |
|  | Neighbors of carcinogenesis |
|  | Neighbors of multiple sclerosis |
|  | Neighbors of chronic inflammation |
|  | Neighbors of inflammation |
|  | Neighbors of pathological angiogenesis |
|  | Neighbors of obesity |
|  | Neighbors of diabetes mellitus |
|  | Neighbors of asthma |
|  | Neighbors of pneumonia |
|  | Neighbors of osteoarthritis |
|  | Neighbors of liver fibrosis |
|  | Neighbors of chronic obstructive pulmonary disease |
|  | Neighbors of myocardial infarction |
|  | Neighbors of leukocyte infiltration |
|  | Neighbors of reperfusion injury |
|  | Neighbors of airway inflammation |
|  | Neighbors of hepatitis |
|  | Neighbors of insulin resistance |
|  | Neighbors of infection |
|  | Neighbors of renal injury |
|  | Neighbors of preeclampsia |
|  | Neighbors of macrophage infiltration |
|  | Neighbors of liver injury |
|  | Neighbors of neuroinflammation |
|  | Neighbors of sepsis |
|  | Neighbors of death |
|  | Neighbors of type 1 diabetes |
|  | Neighbors of encephalomyelitis |
|  | Neighbors of autoimmune disease |
|  | Neighbors of dermatitis |
|  | Neighbors of neutrophil infiltration |
|  | Neighbors of heart disease |
|  | Neighbors of diabetic nephropathy |
|  | Neighbors of lung injury |
|  | Neighbors of immunopathology |
|  | Neighbors of systemic sclerosis |
|  | Neighbors of hepatocellular carcinoma |
|  | Neighbors of autoimmunity |
|  | Neighbors of acute lung injury |
|  | Neighbors of atherosclerotic plaque |
|  | Neighbors of colorectal cancer |
|  | Neighbors of cardiac remodeling |
|  | Neighbors of breast cancer |
|  | Neighbors of ischemia |
|  | Neighbors of systemic lupus erythematosus |
|  | Neighbors of melanoma |
|  | Neighbors of collagen-induced arthritis |
|  | Neighbors of interstitial fibrosis |
|  | Neighbors of kidney disease |
|  | Neighbors of heart failure |
|  | Neighbors of lung disease |
|  | Neighbors of cardiovascular disease |
|  | Neighbors of stroke |
|  | Neighbors of cancer |
|  | Neighbors of type 2 diabetes |
|  | Neighbors of neoplasm |
|  | Neighbors of endometriosis |
|  | Neighbors of neointima |
|  | Neighbors of vasculitis |
|  | Neighbors of atrophy |
|  | Neighbors of diet-induced obesity |
|  | Neighbors of respiratory hypersensitivity |
|  | Neighbors of synovitis |
|  | Neighbors of endothelial cell dysfunction |
|  | Neighbors of liver disease |
|  | Neighbors of prostate cancer |
|  | Neighbors of hypertrophy |
|  | Neighbors of osteolysis |
|  | Neighbors of pancreatic cancer |
|  | Neighbors of hypertension |
|  | Neighbors of intimal hyperplasia |
|  | Neighbors of allograft rejection |
|  | Neighbors of proteinuria |
|  | Neighbors of umbilical vein endothelial cell |
|  | Neighbors of alveolar macrophage |
|  | Neighbors of macrophage |
|  | Neighbors of adipocyte |
|  | Neighbors of pneumocyte |
|  | Neighbors of microglia |
|  | Neighbors of hepatocyte |
|  | Neighbors of dendritic cell |
|  | Neighbors of immunocompetent cell |
|  | Neighbors of myeloid cell |
|  | Neighbors of leukocyte |
|  | Neighbors of insulin-secreting cell |
|  | Neighbors of T-cell |
|  | Neighbors of retina cell |
|  | Neighbors of lung |
|  | Neighbors of liver |
|  | Neighbors of kidney |
|  | Neighbors of lymph node |
|  | Neighbors of placenta |
|  | Neighbors of lymphatic system |
|  | Neighbors of blastocyst |
|  | Neighbors of sympathetic nervous system |
|  | Neighbors of endocrine system |
|  | Neighbors of NF-kB family |
|  | Neighbors of JNK |
|  | Neighbors of IL1 family |
|  | Neighbors of PPAR |
|  | Neighbors of Jun/Fos |
|  | Neighbors of NFKBI |
|  | Neighbors of PKC |
|  | Neighbors of PI3K |
|  | Neighbors of STAT family |
|  | Neighbors of mitogen-activated protein kinase |
|  | Neighbors of IFNAR ligand |
|  | Neighbors of transforming growth factor |
|  | Neighbors of TLR |
|  | Neighbors of cytokine |
|  | Neighbors of interferon |
|  | Neighbors of interleukin |
|  | Neighbors of JAK |
|  | Neighbors of MEK1/2 |
|  | Neighbors of PDGF |
|  | Neighbors of ERK1/2 |
|  | Neighbors of growth factor |
|  | Neighbors of PKA |
|  | Neighbors of ovalbumin |
|  | Neighbors of beta adrenoceptor |
|  | Neighbors of chorionic gonadotropin |
|  | Neighbors of IKBK |
|  | Neighbors of adipokine |
|  | Neighbors of IgG |
|  | Neighbors of SMAD subfamily |
|  | Neighbors of AMPK |
|  | Neighbors of LXR |
|  | Neighbors of p38 |
|  | Neighbors of inflammatory cytokine |
|  | Neighbors of IL23 |
|  | Neighbors of estrogen receptor |
|  | Neighbors of T-cell receptor |
|  | Neighbors of histone deacetylase |
|  | Neighbors of IL1R |
|  | Neighbors of GPCR |
|  | Neighbors of IL12 |
|  | Neighbors of Notch |
|  | Neighbors of heat shock protein 90 |
|  | Neighbors of fibrinogen |
|  | Neighbors of interleukin-35 |
|  | Neighbors of TSH |
|  | Neighbors of Ang II receptor |
|  | Neighbors of endothelin |
|  | Neighbors of proteasome endopeptidase complex |
|  | Neighbors of class nuclear receptor with C4 zinc fingers |
|  | Neighbors of STAT5 |
|  | Neighbors of ITG |
|  | Neighbors of CD3 complex |
|  | Neighbors of TLR1/2 heterodimer |
|  | Neighbors of TORC1 |
|  | Neighbors of histone |
|  | Neighbors of neuropeptides |
|  | New Pathway (3) |
|  | 1\_Differentiation of psoriatic T cells |
|  | 4\_1\_Polymorphisms associated with inflammatory bowel diseases |
|  | 4\_2\_Insulin resistance: inflammation-related insulin resistance |
|  | Differentiation of psoriatic T cells |

---

|  |  |
| --- | --- |
| Group | Genes Hypermethylated in Melanoma |

---

|  |  |
| --- | --- |
| MedScan ID | 9021 |

---

|  |  |
| --- | --- |
| LocusLink ID | 9021 |
|  | 12702 |
|  | 89829 |

---

|  |  |
| --- | --- |
| Alias | E2a-Pbx1 target gene in fibroblasts 10 |
|  | suppressor of cytokine signaling 3 |
|  | STAT induced STAT inhibitor III |
|  | suppressor of cytokine signaling III |
|  | cytokine-inducible SH2 protein III |
|  | SSI-3 |
|  | Dermatitis, atopic, 4 |
|  | Dermatitis, atopic, IV |
|  | suppressor of cytokine inducible signaling 3 |
|  | STAT induced STAT inhibitor 3 |
|  | suppressor of cytokine signaling SOCS3 |
|  | cytokine-induced SH2 protein III |
|  | SSI III |
|  | ATOD4 |
|  | MGC71791 |
|  | Ef10 |
|  | RP23-268N22.6 |
|  | SSI IIIs |
|  | OTTMUSP00000003963 |
|  | cytokine-inducible SH2 protein 3 |
|  | cytokine-induced SH2 protein 3 |
|  | EF X |
|  | cytokine inducible sh2-containing protein 3 |
|  | cytokine inducible sh2-containing protein III |
|  | SOCS3 |
|  | CIS3 |
|  | Cish3 |
|  | SSI3 |
|  | SOCS-3 |
|  | STAT-induced STAT inhibitor 3 |
|  | EF-10 |
|  | CIS-3 |
|  | suppressor of cytokine inducible signalling 3 |

---

|  |  |
| --- | --- |
| GO ID | 0046935 |
|  | 0001784 |
|  | 0004860 |
|  | 0007259 |
|  | 0060670 |
|  | 1990830 |
|  | 0019221 |
|  | 0070102 |
|  | 0035556 |
|  | 0046426 |
|  | 0043066 |
|  | 0050728 |
|  | 0046627 |
|  | 0006469 |
|  | 0042532 |
|  | 0046854 |
|  | 0060674 |
|  | 0045597 |
|  | 0042531 |
|  | 0043687 |
|  | 0016567 |
|  | 0040008 |
|  | 0060334 |
|  | 0043551 |
|  | 0060708 |
|  | 0060707 |
|  | 0005829 |
|  | 0005942 |
|  | 0009968 |
|  | 0045595 |
|  | 0001932 |
|  | 0007165 |
|  | 0005737 |
|  | 0007568 |
|  | 0031100 |
|  | 0001558 |
|  | 0009617 |
|  | 0034097 |
|  | 0042493 |
|  | 0032355 |
|  | 0032094 |
|  | 0010332 |
|  | 0051384 |
|  | 0009408 |
|  | 0009725 |
|  | 0001666 |
|  | 0032868 |
|  | 0032496 |
|  | 0014070 |
|  | 0043434 |
|  | 0032570 |
|  | 0042517 |
|  | 0005622 |
|  | 0060397 |
|  | 0060333 |
|  | 0060338 |
|  | 0060337 |
|  | 0007243 |
|  | 0006916 |

---

|  |  |
| --- | --- |
| KEGG ID | hsa:9021 |
|  | mmu:12702 |
|  | rno:89829 |

---

|  |  |
| --- | --- |
| Organism | Homo sapiens {Organism urn:agi-taxid:9606} |
|  | Mus musculus {Organism urn:agi-taxid:10090} |
|  | Rattus norvegicus {Organism urn:agi-taxid:10116} |
|  | Homo sapiens |
|  | Mus musculus |
|  | Rattus norvegicus |

---

|  |  |
| --- | --- |
| Mouse chromosome position | 11 |

---

|  |  |
| --- | --- |
| OMIM ID | 604176 |
|  | 605805 |

---

|  |  |
| --- | --- |
| Rat chromosome position | 10q32.2 |
|  | 10q32.3 |

---

|  |  |
| --- | --- |
| Hugo ID | 19391 |
|  | HGNC:19391 |

---

|  |  |
| --- | --- |
| Human chromosome position | 17q25.3 |

---

|  |  |
| --- | --- |
| Swiss-Prot Accession | O14543 |
|  | Q6FI39 |
|  | O14543.1 |
|  | O35718 |
|  | O35718.1 |
|  | O88583 |
|  | G3V6D2 |
|  | O88583.1 |
|  | O14509 |
|  | P97803 |
|  | Q3U7X5 |
|  | Q9QYV5 |
|  | B1AQL6 |
|  | Q5U0H7 |
|  | Q3U9J8 |

---

|  |  |
| --- | --- |
| PIR ID | JC5627 |
|  | JC5761 |

---

|  |  |
| --- | --- |
| GenBank ID | NC\_000017 |
|  | NM\_003955 |
|  | NP\_003946 |
|  | NG\_016851 |
|  | AC061992 |
|  | CH471099 |
|  | EAW89521 |
|  | EAW89522 |
|  | AB004904 |
|  | BAA22430 |
|  | AB006967 |
|  | BAA22537 |
|  | AF159854 |
|  | AAD42231 |
|  | AK309756 |
|  | AK314212 |
|  | BAG36886 |
|  | AU134017 |
|  | BC060858 |
|  | AAH60858 |
|  | BE048635 |
|  | BE245649 |
|  | BT019544 |
|  | AAV38351 |
|  | CR536497 |
|  | CAG38736 |
|  | CR541694 |
|  | CAG46495 |
|  | DA671124 |
|  | O14543 |
|  | NC\_000077 |
|  | XM\_011248707 |
|  | XP\_011247009 |
|  | NM\_007707 |
|  | NP\_031733 |
|  | AF117732 |
|  | AAD18024 |
|  | AF314501 |
|  | AAK60601 |
|  | AL591433 |
|  | CH466558 |
|  | EDL34646 |
|  | AK047165 |
|  | BAC32977 |
|  | AK139241 |
|  | BAE23929 |
|  | AK150719 |
|  | BAE29797 |
|  | AK151761 |
|  | BAE30668 |
|  | AK152441 |
|  | BAE31222 |
|  | AK152468 |
|  | BAE31244 |
|  | AK152514 |
|  | BAE31277 |
|  | AK152686 |
|  | BAE31417 |
|  | AK155097 |
|  | AK157708 |
|  | BAE34161 |
|  | AK159395 |
|  | BAE35049 |
|  | AK170406 |
|  | BAE41773 |
|  | AK172399 |
|  | BAE42985 |
|  | BC052031 |
|  | AAH52031 |
|  | U72673 |
|  | AAB51035 |
|  | U88328 |
|  | AAB62403 |
|  | O35718 |
|  | NC\_005109 |
|  | XM\_008768398 |
|  | XP\_008766620 |
|  | NM\_053565 |
|  | NP\_446017 |
|  | AC\_000078 |
|  | AABR07030870 |
|  | AAHX01066870 |
|  | AJ249240 |
|  | CAB56083 |
|  | CH473948 |
|  | EDM06745 |
|  | AF075383 |
|  | AAC26223 |
|  | O88583 |
|  | NC\_018928 |
|  | AMYH02034054 |
|  | AM392896 |
|  | CAL37774 |
|  | AM393146 |
|  | CAL38024 |
|  | AM393491 |
|  | CAL38368 |
|  | AC\_000033 |
|  | AAHY01096850 |
|  | AC\_000149 |
|  | ABBA01002927 |
|  | ABBA01002928 |
|  | XM\_006532110 |
|  | XP\_006532173 |
|  | CAM19105 |
|  | NT\_010783 |
|  | NW\_926918 |
|  | NW\_001838455 |
|  | AC\_000060 |
|  | CR602820 |
|  | Q5U0H7 |
|  | Q6FI39 |
|  | NT\_165773 |
|  | NW\_001030434 |
|  | Q3U9J8 |
|  | NW\_001084656 |
|  | NW\_047344 |

---

|  |  |
| --- | --- |
| Swiss-Prot ID | SOCS3\_HUMAN |
|  | SOCS3\_MOUSE |
|  | SOCS3\_RAT |
|  | B1AQL6\_MOUSE |
|  | Q6FI39\_HUMAN |

---

|  |  |
| --- | --- |
| Cell Localization | Cytoplasm |

---

|  |  |
| --- | --- |
| Ensembl ID | ENSG00000184557 |
|  | ENSP00000330341.2 |
|  | ENST00000330871.3 |
|  | ENSMUSG00000053113 |
|  | ENSMUSP00000059129.3 |
|  | ENSMUST00000054002.3 |
|  | ENSRNOG00000002946 |
|  | ENSRNOP00000003940.1 |
|  | ENSRNOT00000003940.3 |
|  | ENSP00000330341 |
|  | ENST00000330871 |
|  | ENSMUSP00000059129 |
|  | ENSMUST00000054002 |
|  | ENSRNOP00000003940 |
|  | ENSRNOT00000003940 |

---

|  |  |
| --- | --- |
| MGI ID | MGI:1201791 |
|  | 1201791 |

---

|  |  |
| --- | --- |
| RGD ID | 621087 |

---

|  |  |
| --- | --- |
| Unigene ID | Hs.527973 |
|  | Hs.703620 |
|  | Mm.3468 |
|  | Rn.127801 |

---

|  |  |
| --- | --- |
| Homologene ID | 2941 |

---

|  |  |
| --- | --- |
| IPI ID | IPI00210797 |
|  | IPI00297349 |
|  | IPI00133840 |

---
